# Phytoecdysteroids: Quantification in Selected Plant Species and Evaluation of Some Effects on Gastric Smooth Muscles

**DOI:** 10.3390/molecules29215145

**Published:** 2024-10-31

**Authors:** Velislava Todorova, Stanislava Ivanova, Viktor Yotov, Ekaterina Zaytseva, Raina Ardasheva, Valentin Turiyski, Natalia Prissadova, Kalin Ivanov

**Affiliations:** 1 Department of Pharmacognosy and Pharmaceutical Chemistry, Faculty of Pharmacy, Medical University of Plovdiv, 4002 Plovdiv, Bulgaria; 2 Research Institute, Medical University of Plovdiv, 4002 Plovdiv, Bulgaria; 3 Department of Medical Physics and Biophysics, Faculty of Pharmacy, Medical University of Plovdiv, 4002 Plovdiv, Bulgaria

**Keywords:** phytoecdysteroids, 20-hydroxyecdysterone, *Rhaponticum carthamoides* Wild., quinoa, kaniwa, spinach, asparagus, smooth muscle, gastric corpus

## Abstract

Phytoecdysteroids (PEs) are naturally occurring steroid compounds, that have recently gained significant attention, due to their diverse biological activities and high therapeutic potential. The aim of the present study was to quantify some PEs including 20-hydroxyecdysterone (20-HE), ponasterone A (PA), and turkesterone (TU) in selected plant foods and *Rhaponticum carthamoides* extract. Furthermore, the effects of 20-HE, TU, and *R. carthamoides* extract, were investigated with in vitro methods using isolated smooth muscle tissues. The levels of 20-HE in the analyzed samples exhibited significant differences, with kaniwa seed extract containing the highest amount, followed by spinach leaf extract, quinoa seed extract, and asparagus stem extract. The in vitro analyses suggested that *R. carthamoides* extract exhibits dose-dependent cytotoxic effects on smooth muscle cells, with low doses promoting contraction and higher doses inducing relaxation. Additionally, the extract demonstrated a significant inhibitory effect on ACh-induced contractions, while 20-HE enhanced the contractile response. The current findings highlighted phytoecdysteroids’ potential for modifying gastrointestinal motility.

## 1. Introduction

Phytoecdysteroids (PEs) belong to the large family of ecdysteroids. In general, they are plant hormones and secondary metabolites, discovered in many plant species and fungi [1,2,3]. These compounds accumulate in various plant organs, such as fruits, seeds, flowers, leaves, and roots [4,5]. Identified in certain plants, PEs exhibit a significant range of structural diversity [6]. Natural sources of PEs are regarded as plants such as *Cyanotis arachnoidea*, *Cyathula capitata*, *Rhaponticum carthamoides* Wild., *Polypodium vulgare*, *Vitex glabrata*, and *Serratula coronata* [4,7,8].

*Rhaponticum carthamoides* is considered one of the most significant sources of 20-hydroxyecdysterone (20-HE). The plant is associated with a broad spectrum of biological effects including antioxidant, immunomodulatory, antimicrobial, antiparasitic, anticancerogenic, and repellent activities [9,10]. Nowadays, extracts or specific compounds isolated from its roots and rhizomes are included in the composition of various dietary supplements and preparations. They are utilized to support muscle growth, alleviate physical weakness and mental fatigue, and accelerate recovery after surgeries, infectious diseases, or chemical intoxication. *Rhaponticum carthamoides* is not the only source of 20-HE. There are many edible plants that are sources of PEs. It is considered that the inclusion of these plants in the diet of physically active people or people who want to intake PEs will significantly benefit their health condition [9,11,12,13].

Some of the edible plants rich in PEs include spinach (*Spinacia oleracea* L.), white beet (*Beta vulgaris* L.), quinoa (*Chenopodium quinoa* Wild), kaniwa (*Chenopodium pallidicaule* Aellen), asparagus (*Asparagus officinalis* L.), fat hen (*Chenopodium album* L.), and kaki (*Diospyros kaki* Thunb.), etc. [7,14,15]. Particularly rich in 20-HE are quinoa, kaniwa, and spinach from Chenopodiaceae. Quinoa and kaniwa, as gluten-free crops, are nutrient-rich and widely consumed due to their high levels of proteins, amino acids, vitamins, minerals, and dietary fibers [16,17,18,19,20,21,22]. Quinoa contains up to 90% 20-HE from total PE content and exhibits antioxidant, anti-inflammatory and cardio-protective properties, while kaniwa provides antioxidant and anti-anemic effects [16,17,18,19,23,24,25,26,27]. Currently, spinach is regarded as a superfood because of its valuable nutritional profile (it contains beta-carotene, lutein, folate, vitamin C, calcium, iron, phosphorous, potassium, etc.) [28,29,30,31]. Asparagus is another popular and valuable superfood, rich in PEs. It is a low-calorie, nutrient-rich vegetable that has been used as a food and medicine since ancient times. Asparagus contains diverse phytochemicals such as polyphenols, flavonoids, polysaccharides, anthocyanins, and saponins, which are associated with antioxidant, immunomodulatory, hypoglycemic, antihypertensive, antifungal, lipid-lowering and anti-tumor effects [32,33,34,35].

Recently, 20-HE and turkesterone gained significant scientific interest. 20-hydroxyecdysone (ecdysterone) can reach up to 1.5% dry weight of the plant in *R. carthamoides*, while in *Cyanotis arachnoidea* it can reach 4–5% [9,36,37]. Ponasterone A (PA) is another important PE. Initially the compound was isolated from *Podocarpus nakaii*, but it is also found in *R. carthamoides* [9,36,38]. 20-hydroxyecdysone, TU, and PA have been reported to possess anabolic activity, while 20-HE and TU are associated with anti-tumor activity, neuroprotective effects, potential to decrease triglycerides, potential to prevent adiposity, hyperglycemia, and dyslipidemia [6,39,40,41,42,43,44,45].

In 2020, ecdysterone was included in the World Anti-Doping Agency (WADA) monitoring program. This inclusion was associated with its mechanism of action, especially the activation of estrogen receptor beta, although it is still under investigation for its complete biological effect [46,47,48]. Currently, 20-HE is still present in the monitoring program.

However, its inclusion in the Prohibited List of WADA seems to be unlikely because the compound is present in many plant foods.

Although intense research has been conducted about the biological activity of 20-HE, limited data exist about the effects of ecdysteroids on gastrointestinal smooth muscle cells. Further in vitro investigations and cell culture experiments would be especially valuable for better evaluating this molecule’s therapeutic potential. Such studies would enrich the understanding of the mechanisms of action of ecdysteroids on gastrointestinal smooth muscle cells and provide valuable information regarding their potential therapeutic applications.

The aim of the current study was to quantify 20-HE, PA, and TU in various plant extracts and a commercial product containing a liquid extract of *R. carthamoides*. Another key point of this study was the evaluation of the biological activity of 20-HE, TU, and *R. carthamoides* extract through in vitro methods using isolated smooth muscle tissues.

## 2. Results

### 2.1. HPTLC Analysis

In the present study, a previously developed and validated method was utilized for the determination and quantification of 20-HE, PA, and TU [49].

**Figure 1:**
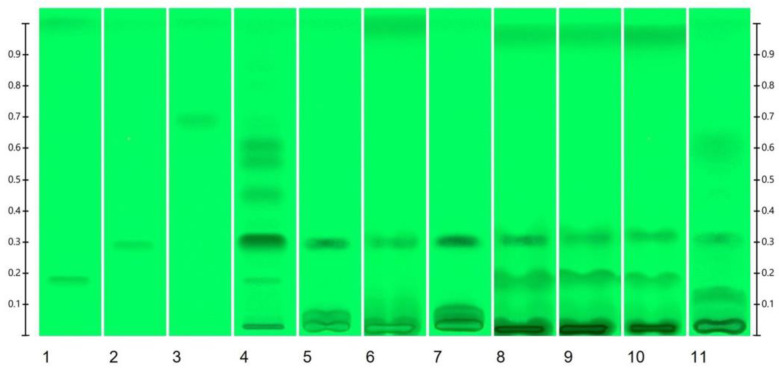
The HPTLC chromatogram of *R. carthamoides* commercial product and prepared plant extracts, where 1. TU 0.75 μg·band^−1^; 2. 20-HE 0.75 μg·band^−1^; 3. PA 0.75 μg·band^−1^; 4. *R. carthamoides* extract commercial product (20 µL); 5. white quinoa seeds extract (20 µL); 6. red quinoa seeds extract (20 µL); 7. kaniwa seeds extract (20 µL); 8. spinach leaves extract S1 (20 µL); 9. spinach leaves extract S2 (20 µL); 10. spinach leaves extract S3 (20 µL); and 11. asparagus stems extract (20 µL).

The prepared extracts (from spinach, quinoa, kaniwa, and asparagus) and *R. carthamoides* commercial product were subjected to HPTLC analysis. The resulting HPTLC chromatogram is shown on Figure 1, and Table 1 presents the results of identification and quantification of 20-HE, PA and TU in the tested samples.

As shown in Table 1, the 20-HE content varied among the different plant samples. The measured concentration of 20-HE in the commercial product of *R. carthamoides* coincides with the label. Among the edible plants, kaniwa was found to contain the highest concentration (670 µg/g dry mass), followed by quinoa (white variety—310 µg/g dry mass and red variety—259 µg/g dry mass), spinach (with values ranging from 252 to 455 µg/g dry mass), and asparagus (189 µg/g dry mass). Due to the limitations of the HPTLC analysis, the obtained results were confirmed using the previously validated HPLC/UV method [11]. The obtained results indicate that 20-HE is the predominant ecdysterone in the observed plants. The presence of PA and TU was negligible or lower than the limit of detection.

For further analyses of biological activity, 20-HE, TU, and the commercial product containing *R. carthamoides* extract were selected. The commercial product was chosen due to higher 20-HE content, compared to the other plant extracts involved in the present study.

### 2.2. Biological Activity

#### 2.2.1. Biological Activity on Primary Smooth Muscle (SM) Culture

A real-time analysis in the xCELLigence system was performed to assess the cells’ viability. The electrical impedance of the cell monolayer was measured continuously and non-invasively, allowing monitoring of changes in cell number, cell size, the extent of cell–cell contacts, and cell-substrate attachment quality. It is worth noting the inter-experimental differences in the dynamics of the cell monolayer development (observable on CI control curves). A probable cause of this may be the work with primary cell culture, the number of cells and the time required for attachment, as well as the rate of cell monolayer development being varied markedly and can hardly be unified. Therefore, the biological effects observed in each experiment are discussed relative to their individual control.

**Figure 2:**
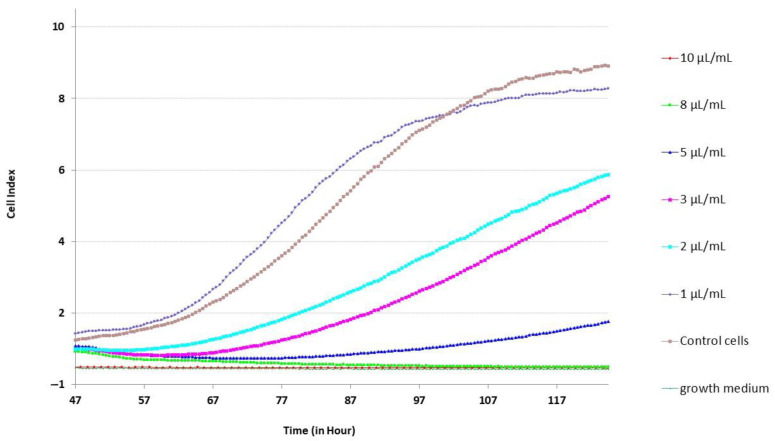
A recording of the time evolution in the cell index after the application of RCE (on the 47th hour) on primary SM (gastric corpus) culture.

A pronounced cytotoxic effect is manifested at high concentrations (10 μL/mL and 8 μL/mL) of *R. carthamoides* extract. In Figure 2, it is observable that the Cell Index (CI) decreased dramatically 10 h after application with the highest *R. carthamoides* extract (RCE) concentration—10 μL/mL growth medium (10 μL/mL). This means that there are no cells attached to the surface, so the viability is considered 0%. *R. carthamoides* extract treatment at the concentration of 8 μL/mL causes the vitality of the cells to decrease significantly (close to zero) 15 h after application.

The data of cell treatment with intermediate concentrations (2, 3 and 5 μL/mL) did not reveal statistically significant effects in the first 10 h after application. Prolonged real-time analysis shows a slowdown in the dynamics of the cellular index. The slope of CI curves, obtained with the RTCA 2.0 software data analysis tool (Table 2), clearly demonstrated decreasing cell proliferation with increasing RCE concentration (relative to the control cells). No significant differences were observed in the CI and the slope between wells treated with the lowest concentrations of 1 μL/mL and control. The dose–response curve generated in the RTCA software was used to determine IC_50_ values. An average IC_50_ value for RCE was calculated at 2.6 ± 1.3 μL of RCE for 1 mL of growth medium for the primary smooth muscle cell culture from rat stomach.

The real-time cell vitality experiment exposed to 20-HE and TU DMSO solutions revealed an acute cytotoxic effect at a dose of 100 µg/mL (100 µL solution per 1 mL growth medium). The sharp decline in cell index is due to a high DMSO content in the growth medium. The same cytotoxic effect is observed when cells are treated with 0.9% DMSO.

**Figure 3:**
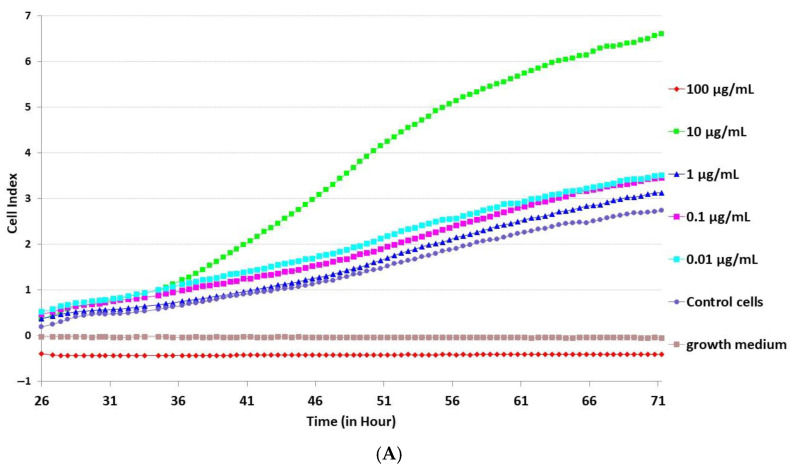

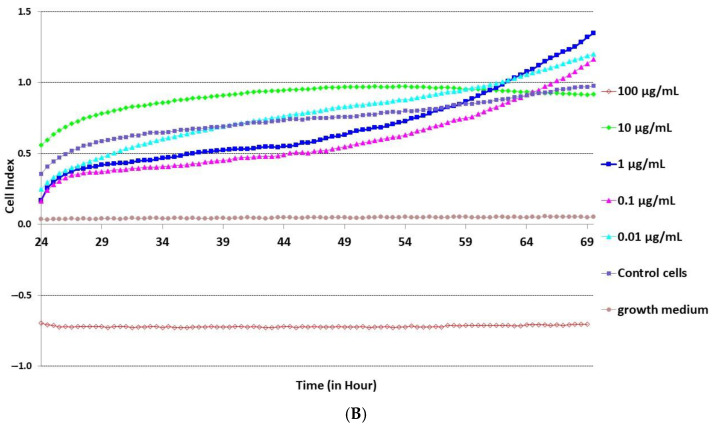
The real-time assay of cell index evolution after treatment with different concentrations (10 µg/mL, 1 µg/mL, 0.1 µg/mL and 0.01 µg/mL) of (**A**) 20-HE and (**B**) TU.

The real-time assay of cell index evolution after treatment with different concentrations (10 µg/mL, 1 µg/mL, 0.1 µg/mL and 0.01 µg/mL) of 20-HE and TU was carried out (Figure 3). The impedance measurement and ANOVA test with post-hoc Games–Howell data indicated the increased cell growth after 10 µg/mL treatment relative to the 1 µg/mL, 0.1 µg/mL and 0.01 µg/mL concentrations of 20-HE. Significant differences between cells exposed to 10 µg/mL 20-HE to other concentrations and control cells prove the slope of the cell index curves data (Table 3).

The dose–response data of TU treatment show a trend to improve cell proliferation for 10 µg/mL concentration, but the difference is not statistically significant. It is worth noting that the treatment with mixed solution 5 µg/mL 20-HE + 5 µg/mL TU revealed a significantly increased cell growth rate compared to the treatment with 10 µg/mL TU and a slower cell growth rate to 10 µg/mL 20-HE treatment. The slope of the cell index curve of the mix solution was calculated as 0.019 ± 0.002 h^−1^, while for TU it was 0.013 ± 0.001 h^−1^ and 0.097 ± 0.032 h^−1^ for 20-HE.

EC_50_ values were estimated with the RTCA 2.0 software data analysis tool for 20-HE (3.4 × 10^−5^ ± 1.5 × 10^−6^ g/mL) and for TU (7.9 × 10^−6^ ± 0.3 × 10^−6^ g/mL).

#### 2.2.2. The Influence of *R. carthamoides* on the Contractile Activity of Gastric SM Preparations

**Figure 4:**
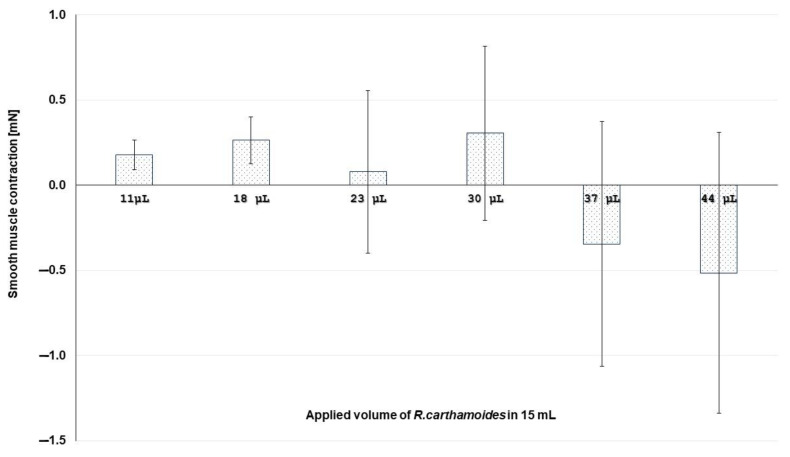
A dose–response graphic of RCE (series of application) on the contraction ability of isolated gastric SM samples (*n* = 6). The negative values present a relaxation phase.

As a result of consecutive exposures to gradually increasing the dose of RCE (series of application—this is the successive application of *R. carthamoides* extract of 11 μL, 18 μL, 23 μL, 30 μL, 37 μL and 40 μL in the tissue bath without washing the sample before and after applications), changes in the contractile activity of isolated circular SM preparations were observed. At low doses, RCE induces a contractile response, which changes to a relaxation response after treatment with higher doses (Figure 4).

#### 2.2.3. Effect of *R. carthamoides* on the Strength of Acetylcholine-Induced Contractions

**Figure 5:**
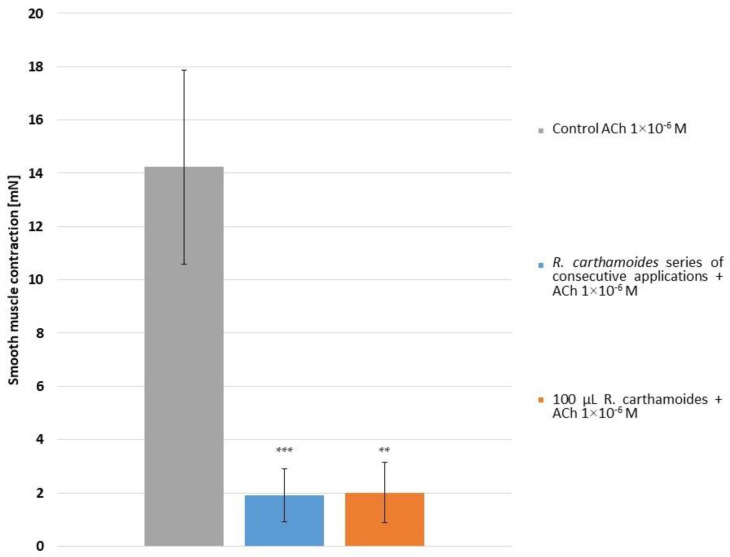
The amplitude of SM (*n* = 6) responses from ACh, series of application of RCE and 100 μL of RCE on the contraction activity of isolated gastric SM samples. ** *p* < 0.01 and *** *p* < 0.001, both compared to the control ACh-induced reaction.

At a concentration of 1 × 10^−6^ mol/L, acetylcholine (Ach) contracted rat stomach smooth muscle preparations (Figure 5). In the indicated dose and series of application, RCE significantly reduced the strength of ACh-induced contractions (*p* < 0.001 and *p* = 0.002, respectively).

#### 2.2.4. Effect of Ecdysterone on the Strength of ACh-Induced Contractions

**Figure 6:**
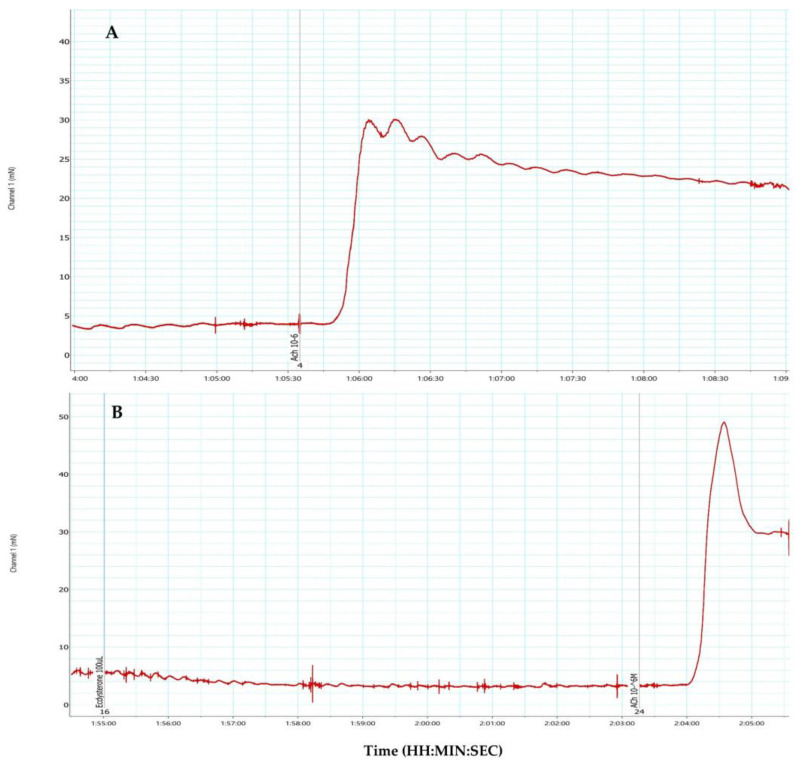
A partial view from the recording of the SM contraction amplitude responses (*n* = 3) induced from 1 × 10^−6^ M ACh (**A**) and a single application of pure 20-HE (100 μL of volume) in the tissue bath and 1 × 10^−6^ M Ach on the background of 100 μL 20-HE (**B**).

The preliminary studies revealed changes in the contractile activity of SM preparations after treatment with 20-HE (Figure 6). The conducted experiments indicated that in the presence of 100 μL 20-HE, the contractile response induced by 10^−6^ mol/L ACh was significantly increased.

## 3. Discussion

A wide variety of analytical methods for the identification, separation, and quantification of PEs, currently exist. However, the HPTLC technique stands out as a convenient and effective approach for qualitative and quantitative analysis, as well as standardization of medicinal materials. HPTLC offers several advantages, such as the ability to analyze multiple samples simultaneously, uncomplicated sample preparation without the need for prior solvent manipulations like filtration or degassing and reduced solvent consumption. Compared to classical Thin-Layer Chromatography (TLC), HPTLC provides enhanced separation efficiencies, minimizes mobile phase usage, enables automation of plate drying, decreases the time required for analysis, and reduces exposure to solvents [50,51].

Using the sensitive HPTLC method for PEs, 20-HE was quantified at 310 µg/g dry mass in quinoa seeds extract, this corresponds to the previously reported range of 20-HE from 138 to 570 µg/g in quinoa seeds [27,52]. Previously, Foucault et al., in 2012 reported that the yield of 500 g of quinoa seeds was 5 g of extract enriched with 1.9% 20-HE, which possesses an anti-obesity effect on mice [53]. A sample with kaniwa seeds extract was found to contain 670 µg/g dry mass, higher than 15 µg/g, as reported previously by Rastrelli et. al., 1996 [54]. The variation in results could potentially be attributed to factors such as regional origin and harvest time, etc. When comparing the three spinach samples (Spinach S1, S2, and S3) based on 20-HE content, the following observations could be made: spinach S1 has the greatest 20-HE content (455 µg/g dry mass). Spinach S2 has a lower content (266 µg/g dry mass), a substantial reduction from S1. Spinach S3 had the lowest level of 20-HE (252 µg/g dry mass), slightly below that of S2. The variations in 20-HE content throughout the three samples could be related to growing conditions, spinach plant age, geographical location, or growth stage. In addition, according to Bakrim et al., the PEs biosynthesis occurs in older leaves, whereas young and developing leaves accumulate them from older leaves. As the plant grows and the number of leaves increases, PEs are constantly redistributed [2]. Regarding spinach, it contained 252–455 µg/g dry mass of 20-HE, aligning with the previously reported range of 79.6 to 428 µg/g, which can vary based on the different processing methods applied to spinach leaves [4]. Bajkacz et al., 2020, reported that the content of 20-HE in spinach leaves can vary between 17.1 and 885 µg/g [55]. The use of 20% ethanol led to an increase in 20-HE content up to 91%, while in the water phase, it remained at approximately 80% [56]. Some authors reported that spinach contained lower amounts of 20-HE, such as 50 μg/g dry mass, 10.3 and 16.8 µg/g according to different spinach accessions, and 0.44 mg% dry weight, respectively [35,57,58]. Grucza et al. reported a lower amount of 20-HE content in fresh spinach leaves—10 µg/100 g [12]. These differences in 20-HE amounts may be due to different processing methods of the spinach leaves before analysis [29]. Moreover, it is considered that in response to mechanical injury or insect damage, spinach accumulates PE [59]. Asparagus stem extract contained 189 µg/g of 20-HE, surpassing the previously reported values of 0.3 µg/g and 2.34 mg% dry weight [35,57]. The content of PA in the *R. carthamoides* commercial product was below the LOQ of the method, and TU was detected at 10.6 µg/mL. None of the prepared plant extracts contained either PA or TU. This finding is consistent with the study by Fang et al. in 2022, which reported that various types of spinach did not contain PA [4].

The current study focused on the effects of *R. carthamoides* extract, 20-HE, and TU on the contractile activity of gastric SM in rats. The effects of the extract are dose dependent and produce a biphasic contraction–relaxation response. A major mechanism in these processes appears to be the activation of signaling cascades associated with protein kinase A (PKA) and protein kinase C (PKC). Elevated intracellular Ca^2+^ levels activate PKC, resulting in SM contraction [60]. Additionally, G-protein-coupled receptors (GPCRs) regulate rapid cellular responses, including the release of Ca^2+^ from the endoplasmic reticulum, increasing intracellular calcium levels [61]. These receptors are expressed differently in smooth muscle cells and interstitial cells of Cajal in the gastrointestinal tract, affecting numerous physiological responses [62]. Research suggests that 20-HE activates GPCRs, resulting in a rapid increase in intracellular Ca^2+^ and subsequent PKC activation, leading to SM contraction [63]. These findings support the notion that PKC activation occurs after increasing intracellular Ca^2+^ levels in response to modest dosages of RCE.

A relationship exists between increasing cytosolic Ca^2+^ levels and adenylate cyclase (AC) activity, which involves a Ca^2+^-dependent enzyme [64]. Enhanced AC activity activates protein kinase A (PKA), leading to SM relaxation. A rapid rise in cytosolic Ca^2+^ levels induced by 20-HE activated AC and PKA, contributing to SM relaxation. This pathway likely explains the relaxation observed at higher RCE doses. The biphasic effect results from a complex mechanism involving PKA activation and 20-HE action on estrogen receptors. Phytoecdysteroids influence signal transduction through membrane-bound receptors, particularly estrogen receptors (alpha and beta) [65,66]. While both are present in mammals, beta receptors dominate in the gastrointestinal tract [67]. Ecdysteroids also induce non-genomic signaling processes that enhance cell proliferation and vitality. Specifically, 20-HE activates secondary signals, including altered Ca^2+^ flux, leading to Akt phosphorylation and increased protein synthesis [8,63]. The observed biphasic effect from the current experiment may result from a complex mechanism involving PKA activation and 20-HE action on estrogen receptors. These data support the significant increase in contractile response induced by 1 × 10^−6^ mol/L ACh in the presence of 20-HE, and the enhanced cellular proliferation in gastric smooth muscle cell cultures.

xCELLigence analysis demonstrated that high concentrations (10 µL/mL and 8 µL/mL) of commercial *R. carthamoides* extract cause cell death in primary cultures of rat stomach smooth muscle cells, whereas lower concentrations inhibit cell proliferation compared with controls. This dose-dependent effect is not likely caused by direct cytotoxicity to cell membranes or essential cellular components. In addition, certain phenolic compounds in *R. carthamoides* extract may play a role in this cytotoxicity. For example, isolated polyphenols (anthocyanins, kaempferol, and quercetin) inhibit the growth of various human tumor cell lines in a dose-dependent manner, with varying sensitivities [68,69].

The minimal impact of TU on cell growth and vitality compared with 20-HE can be attributed to the differences in the hydroxyl groups; TU has an additional hydroxyl group at the C-11 position. The anabolic effect of these compounds is influenced by the presence of diol groups at the C-20 and C-22 positions, whereas an additional hydroxyl group at C-11 significantly enhances protein synthesis [6].

Studies suggest that *R. carthamoides* extracts may play a beneficial role in the treatment of gastrointestinal disorders by modulating gastric smooth muscle contractility [70]. Clinical studies with athletes from the Soviet Union and Russia have shown that *R. carthamoides* extracts, especially those rich in 20-HE, positively influence protein synthesis and increase work capacity [71]. These benefits may also extend to rehabilitation processes following traumatic injury.

The findings of this study are valuable due to limited data on the in vitro effects of *R. carthamoides*, 20-HE, and TU on isolated gastric smooth muscle preparations. The therapeutic potential of *R. carthamoides* and its ecdysteroids is highlighted, supporting their use as nutraceuticals. However, further investigation is required to obtain a deeper understanding of the specific cellular mechanisms of action.

## 4. Materials and Methods

### 4.1. Reference Standards and Chemicals

The standards of 20-hydroxyecdysterone, purity HPLC ≥ 95% and turkesterone, purity HPLC ≥ 95% were obtained from PhytoLab GmbH & Co. KG, Vestenbergsgreuth, Germany, while ponasterone A, purity ≥ 95% was obtained from Cayman Chemical, Ann Arbor, MI, USA. Methanol and acetonitrile were of analytical grade and were purchased from Merck, Darmstadt, Germany. Phosphate-buffered saline (PBS, cat # D8662), antibiotics (penicillin, streptomycin, amphotericin B), collagenase D (cat # 11088858001), trypsin (T2610), Dulbecco’s Modified Eagle Medium (DMEM, cat # D5796), Fetal Bovine Serum (FBS, cat # F7524) and antibiotics (cat # A5955), were purchased from Sigma-Aldrich, St. Louis, MO, USA.

### 4.2. Sample Preparation

Fresh spinach leaves for sample 2 (S2) and sample 3 (S3) (100 g), asparagus stems (100 g), quinoa seeds (from white and red variety) (100 g), kaniwa seeds (5 g), were purchased from local bio markets, and fresh spinach leaves for sample 1 (S1) were collected in Belashtitsa, Thracian Lowland floristic region, Bulgaria, with coordinates 42.064729, 24.751886. They were dried at 60 °C in a dryer (Memmert GmbH Co. KG, Schwabach, Germany). Subsequently, they were ground in a mortar with a pestle and transferred for preparation of the 50% methanolic plant extracts; the extracts were prepared in ultrasonic (Bandelin, Berlin, Germany) for 30 min. The resulting extracts were filtered through a 0.45 µm membrane filter and stored in brown vials at 4 °C, prior to use. The *R. carthamoides* extract was purchased from a pharmacy, and it was labeled as containing 30 mg/mL 20-HE.

### 4.3. HPTLC Analysis

For the HPTLC analysis of plant extracts, the previously developed and validated HPTLC method for PEs was used—20-HE, TU and PA [49]. The method was developed using a CAMAG HPTLC system (CAMAG, Muttenz, Switzerland). CAMAG Limomat 5, (CAMAG, Muttenz, Switzerland) was used as the sample applicator. The HPTLC glass plates (plate size: 10 × 20 cm^2^) pre-coated with normal-phase silica gel with 200 µm were used (Merck, Darmstadt, Germany). The CAMAG 100 µL Syringe (Hamilton, Bonaduz, Switzerland) was attached with the sample applicator. The application type was a band, and the front was 70 mm. CAMAG Automatic Developing Chamber 2 (CAMAG, Muttenz, Switzerland) was used for the developing of the plates. The mobile phase comprised methanol/acetonitrile at a ratio of 10:90 (*v*/*v*), and volume of 10 mL as the mobile phase. The time for development was 10 min, followed by drying of 5 min. Detection was performed at 254 nm using CAMAG TLC Visualizer 2 (CAMAG, Muttenz, Switzerland) which recorded the chromatogram. The software used was “VisionCATS” (version 3, CAMAG, Muttenz, Switzerland). The method was previously validated following the ICH guidelines (good linearity, accuracy, precision and robustness) [49].

### 4.4. Smooth Muscle Activity—Animals, Tissues and Preparations

All experiments were carried out according to the European Union (directive 2010/63/EU) and Bulgarian guidelines (directive No. 20/01.11.2012) for using laboratory animals (License No. 213/5.10.2018) from the Animal Health and Welfare Directorate of the Bulgarian food safety agency [72].

#### 4.4.1. Primary Smooth Muscle Cell Culture: Cells Cultivation

Two male Wistar rats with body weights of 250 g and 280 g were provided by the Animal House of Medical University—Plovdiv, Bulgaria. The rats were bred in standard laboratory conditions (23–25 °C, 50–55% humidity and 12/12 h light/dark cycle). They had ad libitum access to food and water and were deprived of food for 24 h before euthanasia.

Decapitation of the animals was performed after anesthesia with xylazine 10 mg/kg and ketamine 100 mg/kg. Smooth muscle samples from the gastric corpus were collected in situ without the mucosa. During sample collection, the tissue was constantly washed with a sterile saline solution. Immediately after resection, the samples were placed in sterile containers filled with sterile saline. Subsequent procedures were carried out in a laminar flow box, using sterile techniques.

The fragments of the smooth muscle layers were placed in a Petri dish (60 mm diameter) containing phosphate-buffered saline supplemented with antibiotics (100 U/mL penicillin, 100 μg/mL streptomycin, 5 μg/mL amphotericin B) and cut into small pieces (1 mm^2^). Next, the fragments were placed in 1 mg/mL collagenase D and 0.25% trypsin and incubated at 37 °C for 2 h.

After a prolonged digestion time, the enzymes were neutralized by adding a medium containing FBS, and the suspensions were centrifuged at 1000× *g* for 10 min. The cell pellet was resuspended in culture medium. The number of isolated cells was estimated using the trypan blue exclusion test.

The isolated cells were seeded at a density of 2 × 10^4^ cells/cm^2^. The growth media were DMEM supplemented with 20% FBS and antibiotics. Cells were cultured at 37 °C in 5% CO_2_ and 95% humidity. The growth medium was changed every 2–3 days. Evaluation of cell morphology and growth was performed under an inverted light microscope. Cells in these cultures were elongated, spindle-shaped, bipolar with a centrally located nucleus, and exhibited characteristics for the smooth muscle cells’ “hills and valleys” growth pattern. Intensive cell divisions allowed confluence to be reached in 4–5 days of culture. Cell cultures that reached 70–90% confluence and exhibited morphology typical for smooth muscle cells were considered successful.

Cell growth and cell treatment (*R. carthamoides* ethyl alcohol extract—commercial product. The manufacturer states that the concentration of ethyl alcohol in 10 drops or 0.5 mL is 0.169% of the volume. 20-HE-DMSO solution or TU-DMSO solution) were monitored in real-time using the xCELLigence RTCA-DP system (ACEA Biosciences, Agilent, Santa Clara, CA, USA). Cell cultures were trypsinized with 0.25% trypsin and 0.02% EDTA in PBS and the number of cells was measured. After that, they were seeded on a 16-well plate (E-Plate VIEW 16, ACEA Biosciences, Agilent, Santa Clara, CA, USA, cat # 300600880) at a density of 2 × 10^4^ cells/cm^2^ and placed in the xCELLigence system.

For the treatment with the used substances, the plates were removed and the growth medium in the wells was replaced with a growth medium containing different concentrations of *R. carthamoides* extract, 20-HE or TU. The xCELLigence RTCA-DP system transforms the measurement of the electrical impedance (changes in electrical resistance) of adherent cells to Cell Index (CI). The baseline of the CI is defined as the value of the electrical impedance of the growth medium. Measurements were made every 30 min, for 3–4 days. The cell growth curve was plotted as a dependence on the CI from the time of culture. The curves were used to determine IC_50_ or EC_50_ values.

#### 4.4.2. Investigation of Spontaneous Contractile Activity (SCA) and Reactivity of Isolated Smooth Muscles (SM)

Smooth muscle (SM) preparations were isolated from six male Wistar rats (*n* = 6). Immediately after decapitation of the animals, SM preparations from the gastric corpus were cut in situ, separating only the muscular tissue without the mucosa. During the preparation of the specimens, continuous rinsing of the SM tissue with a chilled preparation solution (NaCl—120 mmol/L: KCl—5.9 mmol/L: CaCl_2_—2.5 mmol/L in a ratio of 27.2:1.1:1) was ensured. The circular stomach strips used to record contractile activity had lengths of 15–18 mm and widths of 1.0–1.1 mm.

Smooth muscles preparations were placed in a tissue bath with Krebs’ solution (pH = 7.4, T = 37 °C) containing the following (in mmol/L): NaCl—120; KCl—5.9; CaCl_2_—2.5; MgCl_2_—1.2; NaH_2_PO_4_—1.2; NaHCO_3_—15.4; and glucose—11.5. All chemicals used in the solution were obtained from Merck, USA. The Krebs’ solution was continuously saturated with a mixture of O_2_: CO_2_ in a ratio of 19:1 (*v*:*v*).

The preparations were firmly attached to a glass holder at one end and connected via surgical thread at the other to a force transducer, which was part of the Tissue Organ Bath System (159920, Radnoti, AD Instruments Limited, Oxford, UK). The electrical signal obtained from them was amplified using an amplifier Octal Bridge Amp (FE228, Radnoti, AD Instruments Limited, UK). The recording of mechanical activity was carried out using a PowerLab data acquisition system 8/35 and LabChart analysis software, ver. 8.1.16 (both developed by AD Instruments Limited, UK).

The registration of mechanical activity was performed using the isometric method. The initial mechanical loading for the preparations were achieved by stretching which corresponded to a tension force of 10 mN. The adaptation period for establishing the baseline muscle tone and regular SCA was 60 min. Changes in spontaneous mechanical activity and tone caused by altered conditions or exposure to different biologically active substances were recorded relative to the corresponding initial value.

The effect of the investigated substances on contractile activity was ensured by adding a precisely determined volume of a concentrated solution of the respective substance necessary to achieve the desired concentration in the tissue bath. The volume did not exceed 1/100 part of the volume of the solution in the tissue bath. The vitality of the SM tissues was tested by exposure to 1 × 10^−6^ mol/L acetylcholine (ACh) at the beginning and end of each experiment twice and at the end of each exposure to the investigated substances/conditions.

### 4.5. Statistical Methods

All statistical analyses were performed using SPSS software, version 17.0 (SPSS Inc. Chicago, IL, USA). The analysis began with determination of data distribution with the Shapiro–Wilk normality test. The data obtained are expressed as mean ± standard deviation. Statistical differences were tested using paired t-test and ANOVA (post-hoc Games–Howell test). The probability of *p* < 0.05 was considered significant.

## 5. Conclusions

This study provides data about the levels of 20-HE, PA, and TU in different superfood plant extracts. The analyses were performed using the HPTLC technique. The results suggested that quinoa (310 µg/g 20-HE in dry mass), spinach (252 to 455 µg/g 20-HE in dry mass) and kaniwa (670 µg/g 20-HE in dry mass) could be regarded as good sources of 20-HE. The inclusion of these foods in the diet of physically active people or people who are exposed to stress conditions would definitely beneficial. Although in the last few years, many studies were focused on the biological activity of *R. carthamoides* and 20-HE, the data about their effects on the gastrointestinal smooth muscle cells are limited. An important key point of this study was the evaluation of the biological activity of 20-HE, TU, and *R. carthamoides* extract through in vitro methods using isolated smooth muscle tissues. The results suggested a pronounced cytotoxic effect of *R. carthamoides* extract at doses of 10 μL and 8 μL, and a significant reduction in ACh-induced contraction. Furthermore, it was established that 20-HE at concentrations of 10 μg/mL produced a proliferative effect on the primary gastric smooth muscle cell culture.

## Figures and Tables

**Table 1:** Content of selected PEs (20-HE, PA, and TU) in *R. carthamoides* extract commercial product and plant extracts, where ND—not detected, and LOQ—limit of quantification.

Product/Plant Extract	20-Hydroxyecdysterone	Ponasterone A	Turkesterone
Commercial product	28.2 mg/mL	<LOQ	10.6 µg/ mL
White quinoa	310 µg/g dry mass	ND	ND
Red quinoa	259 µg/g dry mass	ND	ND
Kaniwa	670 µg/g dry mass	ND	ND
Spinach S1	455 µg/g dry mass	ND	ND
Spinach S2	266 µg/g dry mass	ND	ND
Spinach S3	252 µg/g dry mass	ND	ND
Asparagus	189 µg/g dry mass	ND	ND

The results present the mean values for the three independent samples of each product and extract. The standard error of the mean does not exceed 2% and has been omitted to simplify the results.

**Table 2:** Slope of the time evolution in the cell index after the application of RCE (in different concentrations) on primary SM (gastric corpus) culture.

Concentration [μL/mL]	Mean ± SD [h^−1^]
10	−0.002 ± 0.002
8	−0.005 ± 0.001
5	0.011 ± 0.001
3	0.061 ± 0.007
2	0.091 ± 0.009
1	0.127 ± 0.009
Control	0.136 ± 0.005

**Table 3:** Slope of the time evolution in the cell index after the application of 20-HE and TU DMSO solutions (in different concentrations) on primary smooth muscle (gastric corpus) culture.

Concentration [mg/mL]	20-Hydroxyecdysone	Turkesterone
	Mean ± SD [h^−1^]	Mean ± SD [h^−1^]
100	0.001 ± 0.0002	0.002 ± 0.0003
10	0.097 ± 0.032 *	0.013 ± 0.001
1	0.033 ± 0.007	0.035 ± 0.008
0.1	0.043 ± 0.016	0.025 ± 0.004
0.01	0.029 ± 0.018	-
Control	0.036 ± 0.015	0.017 ± 0.003

Note: * *p* < 0.05 compared to the control.

## Data Availability

The original contributions presented in the study are included in the article, further inquiries can be directed to the corresponding author.
